# Enhancing the data capture of periprosthetic joint infections in the Danish Knee Arthroplasty Registry: validity assessment and incidence estimation

**DOI:** 10.2340/17453674.2024.40358

**Published:** 2024-04-09

**Authors:** Marie ANNEBERG, Eskild Bendix KRISTIANSEN, Anders TROELSEN, Per GUNDTOFT, Henrik Toft SØRENSEN, Alma B PEDERSEN

**Affiliations:** 1 Department of Clinical Epidemiology, Aarhus University Hospital, Aarhus University; 2 Department of Orthopedic Surgery, Hvidovre Hospital; 3 Department of Orthopedic Surgery, Aarhus University Hospital; 4 Department of Clinical Medicine, Aarhus University, Aarhus, Denmark

## Abstract

**Background and purpose:**

Revisions due to periprosthetic joint infection (PJI) are underestimated in national arthroplasty registries. Our primary objective was to assess the validity in the Danish Knee Arthroplasty Register (DKR) of revisions performed due to PJI against the Healthcare-Associated Infections Database (HAIBA). The secondary aim was to describe the cumulative incidences of revision due to PJI within 1 year of primary total knee arthroplasty (TKA) according to the DKR, HAIBA, and DKR/HAIBA combined.

**Methods:**

This longitudinal observational cohort study included 56,305 primary TKAs (2010–2018), reported in both the DKR and HAIBA. In the DKR, revision performed due to PJI was based on pre- and intraoperative assessment disclosed by the surgeon immediately after surgery. In HAIBA, PJI was identified from knee-related revision procedures coinciding with 2 biopsies with identical microbiological pathogens. We calculated the sensitivity, specificity, positive predictive value (PPV), and negative predictive value (NPV) of revision due to PJI in the DKR (vs. HAIBA, within 1 year of TKA) with 95% confidence intervals (CI). Cumulative incidences were calculated using the Kaplan–Meier method.

**Results:**

The DKR‘s sensitivity for PJI revision was 58% (CI 53–62) and varied by TKA year (41%–68%) and prosthetic type (31% for monoblock; 63% for modular). The specificity was 99.8% (CI 99.7–99.8), PPV 64% (CI 62–72), and NPV 99.6% (CI 99.6–99.7). 80% of PJI cases not captured by the DKR were caused by non-reporting rather than misclassification. 33% of PJI cases in the DKR or HAIBA were culture-negative. Considering potential misclassifications, the best-case sensitivity was 64%. The cumulative incidences of PJI were 0.8% in the DKR, 0.9% in HAIBA, and 1.1% when combining data.

**Conclusion:**

The sensitivity of revision due to PJI in the DKR was 58%. The cumulative incidence of PJI within 1 year after TKA was highest (1.1%) when combining the DKR and HAIBA, showing that incorporating microbiology data into arthroplasty registries can enhance PJI validity.

Periprosthetic joint infection (PJI) is a rare but serious complication, leading to a revision in 1–4% of patients following total knee arthroplasty (TKA) [1-4]. The data quality of national registries is generally considered high [5-7] and they have been used to advance clinical practice [8].

However, arthroplasty registries are prone to underestimate revisions due to PJI [9-12]. This raises concern as to whether the current identification strategy for revision due to PJI employed by the arthroplasty registries is adequately robust [11].

In the PJI guidelines [13] from 2021, a diagnosis of PJI in the hip or knee is based mainly on the results of microbiology tests in addition to a clinical and paraclinical evaluation. However, microbiology test results are mostly not recorded in arthroplasty registries [1,14] as surgeons report the revision cause to the register immediately after the revision procedure when the biopsy test results are not yet known. Previous studies of PJI based solely on arthroplasty registries identify the lack of microbiology data as a limitation [14,15].

In Denmark, all microbiology test results are recorded in the Danish Microbiology Database (MiBa) [16]. Based on data from MiBa and the Danish National Patient Registry (DNPR) [17], an automated surveillance system has been established—the Healthcare-Associated Infections Database (HAIBA) [18]—using an automated algorithm to register continuous incidence data for specific infectious diseases, including revision due to PJIs after TKA. We hypothesized that by individual-level linkage of the clinically relevant data from the Danish Knee Arthroplasty Register (DKR) [19] and microbiological data from HAIBA, it would be possible to improve the validity of the registration of revision due to PJI in DKR.

Our primary objective was to assess the validity of revision due to PJI registration in the DKR against HAIBA as a reference standard, and additionally to outline the features of the revision due to PJI cases identified by HAIBA but not captured by the DKR. Our secondary objective was to describe the cumulative incidence of revision due to PJI within 1 year of primary TKA according to the DKR, HAIBA, and DKR/HAIBA combined.

## Methods

### Study design, setting, and data sources

We conducted this combined validation and longitudinal observational cohort study using data from the Danish Civil Registration System (CRS) [20], the DNPR, the DKR, MiBa, and the algorithm from HAIBA. Denmark is a country of 5.8 million residents (2018) with tax-supported free access to healthcare [21].

The CRS assigns a Civil Personal Registration (CPR) number to each Danish resident at birth or upon immigration. Denmark has a long tradition of establishing nationwide medical databases, linkable at an individual level via the CPR number. The CRS contains information on migration and the vital status of the entire Danish population since 1968.

The DNPR records data on all inpatient admissions to Danish hospitals since 1977 and all outpatient clinic and emergency room visits since 1994. As hospitals are funded based on Diagnosis Related Group tariffs [22], they report all procedure codes (the Danish version of the Nordic Medico-Statistical Committee [NOMESCO] Surgical procedures) and discharge diagnosis (International Classification of Diseases [ICD]) codes assigned to each hospital contact with the DNPR.

The DKR was established in 1997 and collects data on primary and revision arthroplasties from all Danish public and private orthopedic departments performing knee arthroplasties (23 public and 14 private hospitals in 2018). Surgeons submit procedural data including surgery date to the DKR immediately after performing surgery. When preparing the annual reports, the DKR is validated against the DNPR [23] for patient registration completeness (i.e., whether all patients undergoing knee arthroplasty are registered in the DKR). In 2010, patient registration completeness was 92% for primary knee arthroplasties (including both TKA and unicompartmental knee arthroplasties) and 89% for revisions. By 2018, the completeness had improved, reaching 97% for primary knee arthroplasties and 94% for revisions.

MiBa is a medical database that, since 2010, has collected an electronic copy of all reports from all Danish microbiological departments. The test results are systematically presented for each sample with findings from 1 to x without providing interpretations regarding pathogenicity or contamination. Based on data from MiBa and the DNPR, an automated surveillance system has been established—HAIBA, providing publicly available continuous incidence data for specific infectious diseases, including revisions due to PJI after TKA. To identify PJI revisions, HAIBA retrieves data on primary TKAs, and subsequent knee-related revision procedures as registered in the DNPR (codes provided in Table 1, see Appendix) and links the cases to microbiology test results from MiBa. HAIBA’s algorithm tallies the count of positive samples for each finding within a period ranging from 24 hours before to 48 hours after the knee-related revision procedure. The algorithm considers the results of tissue samples, biopsies, bone tissue samples, Kamme–Lindberg biopsies [24], and joint capsule tissue samples. To classify a revision as attributable to PJI, a minimum of 3 samples must be present within this defined period, with at least 2 of them testing positive for the same bacteria.

**Table 1:** Codes used to define the study cohort, revisions in the DKR and HAIBA, and PJI in the DKR and HAIBA

**Study population**
Primary TKA surgery (index surgery) in a public or private hospital in Denmark during the study period from January 1, 2010 to December 31, 2018, as reported in:
**DKR**: with TKA codes (n = 59,802 TKAs)
**HAIBA**: with NOMESCO Codes (from the DNPR) KNGB20, KNGB30, KNGB40 (n = 66,591 TKAs)
**Any revision**
**DKR**: A revision is defined as any later procedure that involves a supplement to, exchange, removal, or modification of an already existing arthroplasty [20]. All revisions recorded in the DKR (due to aseptic loosening, pain without loosening, instability, deep infection (verified by microbiology), deep infection (suspected), secondary insertion of patellar component, wear of polyethylene (patella), wear of polyethylene (tibia), progression of osteoarthritis, and other less frequent causes).
**HAIBA**: NOMESCO codes from the DNPR (translated from Danish to English).
KNGA02A: Open exploration of soft tissues in the knee
KNGA12: Open exploration of the knee joint
KNGA22A: Open joint biopsy in the knee
KNGA22C: Open soft tissue biopsy in the knee
KNGC20: Secondary insertion of all components of uncemented total knee prosthesis
KNGC21: Secondary insertion of proximal components of uncemented total knee prosthesis
KNGC22: Secondary insertion of distal components of uncemented total knee prosthesis
KNGC23: Secondary insertion, patellofemoral component, uncemented total knee prosthesis
KNGC29: Secondary insertion of uncemented total knee prosthesis, unspecified
KNGC30: Secondary insertion of all components of hybrid total knee prosthesis
KNGC31: Secondary insertion of proximal components of hybrid total knee prosthesis
KNGC32: Secondary insertion of distal components of hybrid total knee prosthesis
KNGC33: Secondary insertion, patellofemoral component, hybrid total knee prosthesis
KNGC39: Secondary insertion of hybrid total knee prosthesis, unspecified
KNGC40: Secondary insertion of all components of cemented total knee prosthesis
KNGC41: Secondary insertion of proximal components of cemented total knee prosthesis
KNGC42: Secondary insertion of distal components of cemented total knee prosthesis
KNGC43: Secondary insertion, patellofemoral component, cemented total knee prosthesis
KNGC49: Secondary insertion of cemented total knee prosthesis, unspecified
KNGC59: Secondary insertion of interpositional prosthesis in the knee joint
KNGC99: Other secondary insertion of knee joint prosthesis
KNGF02: Open total synovectomy in the knee joint
KNGF12: Open partial synovectomy in the knee joint
KNGG09: Resection arthroplasty in the knee
KNGG19: Interpositional arthroplasty in the knee
KNGG29: Other arthroplasty without prosthesis in the knee
KNGG49: Arthrodesis with internal fixation in the knee
KNGG59: Arthrodesis with external fixation in the knee
KNGH32: Open release of adhesions in the knee joint
KNGH92: Other open knee joint surgery
KNGK29: Fenestration or perforation of bone in the knee/leg
KNGM79: Excision of a bursa in the knee/leg
KNGQ09: Disarticulation of the knee joint
KNGQ99: Other amputation surgery on the knee/leg
KNGS19: Incision and revision of infection in the knee joint
KNGS29: Incision/revision of bone infection in the knee/leg
KNGS49: Incision/revision with the installation of drugs for infection in the knee joint
KNGS59: Incision/revision/installation of drugs for bone infection in the knee/leg
KNGS99: Other operations for infection in the tendon, joint, or bone in the knee/leg
KNGU10: Removal of all components of total knee prosthesis
KNGU11: Removal of the medial part of total knee prosthesis
KNGU12: Removal of the lateral part of total knee prosthesis
KNGU19: Removal of total knee prosthesis, unspecified
KNGU99: Removal of another implant in the knee/leg
KNGW59: Reoperation for superficial infection after surgery on the knee/leg
KNGW69: Reoperation for deep infection after surgery on the knee/leg
KNGW89: Reoperation for deep hemorrhage after surgery on the knee/leg
KNGW99: Other reoperation after surgery on the knee/leg
**Periprosthetic joint infection**
**DKR**: Deep infection (verified by microbiology). Deep infection (suspected).
**HAIBA**: From MiBa: Samples considered among patients with any revision: tissue, biopsy, bone, Kamme–Lindberg biopsies, and tissue from the joint capsule. Cultures taken between 24 hours before revision surgery and 48 hours after revision surgery if a minimum of 2 samples out of a minimum of 3 samples were positive with the same microorganism. If only 1/5 of biopsies were positive or 2/5 with different pathogens, it was considered contamination.

Abbreviations: see Table 2.

### Primary TKA cohort

We included patients who underwent primary TKAs (index surgery) in a public or private hospital in Denmark during the study period (January 1, 2010, to December 31, 2018), as reported in the DKR or the DNPR. Patients treated with unicompartmental knee arthroplasties were not included, as HAIBA only monitors TKA procedures.

**Figure 1:**
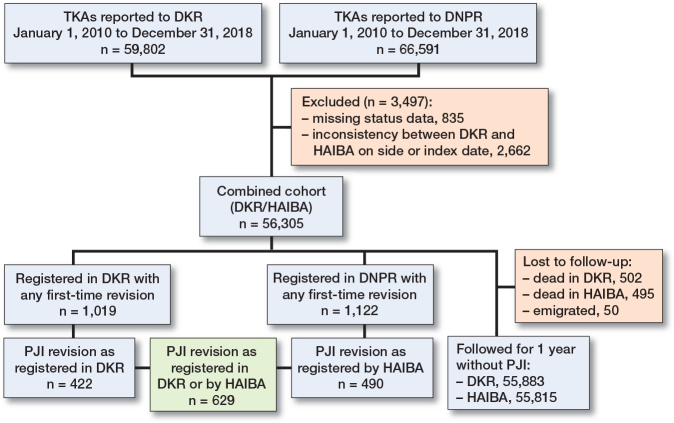
Flowchart. For abbreviations, see footnote Table 2.

Patients were excluded if there was an inconsistency in the date of primary TKA or laterality (Figure 1). If a patient had TKA on both knees, they were included as 2 separate cases.

### Outcome

#### Identification of first-time revision due to any cause

A primary TKA was defined as registered with the same primary surgery date and laterality in the DNPR and the DKR. It was directly linked to the first same-side, knee-related revision surgery occurring within 1 year after the index surgery, henceforth referred to as “any revision.” In the DKR, a revision is defined as any later procedure involving supplementing, exchanging, removing, or modifying an already performed arthroplasty as registered by the surgeon [19]. This encompassed DAIR (debridement, antibiotics, and implant retention) if it included an exchange of liner. In the case of DAIR without any exchange of components (in monoblock prostheses with no exchangeable liner), registration with the DKR is not mandatory. In HAIBA, a revision was defined as any same-side knee-related revision within 1 year of a primary TKA using the procedure codes from the DNPR provided in Table 1 (see Appendix). In the case of a multi-stage revision, we included only the first-stage procedure.

**Table 2:** Validity of data on revision due to PJI in the DKR with HAIBA data as the reference standard within 3 months and 1 year of primary TKA. Values are counted with percentage of the total or percentage with a 95% confidence interval unless otherwise specified

Factor	Total TKAs	True positive [a]	False positive [b]	False negative [c]	True negative [d]	Sensitivity a/(a+c)	Specificity d/(b+d)	Positive predictive value a/(a+b)	Negative predictive value d/(c+d)
Age, median	69	70	73	74	69				
(IQR)	(62–75)	(62–77)	(68–79)	(66–81)	(62–75)				
BMI, median	28.9	31.6	28.4	29.6	28.9				
(IQR)	(26–33)	(27–35)	(24–33)	(26–34)	(26–33)				
**Stratified analyses for revision due to PJI within**									
1 year	56,305 (100)	283 (0.5)	139 (0.2)	207 (0.4)	55,676 (99)	58 (53–62)	99.8 (99.7–99.8)	67 (62–72)	99.6 (99.6–99.7)
3 months	56,305 (100)	204 (0.4)	68 (0.1)	183 (0.3)	55,850 (99)	53 (48–58)	99.9 (99.9–99.9)	75 (69–80)	99.7 (99.6–99.7)
1 year after primary TKA by year of primary TKA									
2010	6,360 (11)	25 (0.4)	13 (0.2)	36(0.6)	6,286 (99)	41 (29–54)	99.8 (99.6–99.9)	66 (49–80)	99.4 (99.2–99.6)
2011	6,355 (11)	42 (0.7)	23(0.4)	24(0.4)	6,266 (99)	64 (51–75)	99.6 (99.5–99.8)	65 (52–76)	99.6 (99.4–99.8)
2012	6,538 (12)	28 (0.4)	15 (0.2)	13 (0.2)	6,482 (99)	68 (52–82)	99.8 (99.6–99.9)	65 (49–79)	99.8 (99.7–99.9)
2013	6,572 (12)	34 (0.5)	12 (0.2)	22 (0.3)	6,504 (99)	61 (47–74)	99.8 (99.7–99.9)	74 (59–86)	99.7 (99.5–99.8)
2014	6,699 (12)	27 (0.4)	14 (0.2)	28 (0.4)	6,630 (99)	49 (35–63)	99.8 (99.6–99.9)	66 (49–80)	99.6 (99.4–99.7)
2015	6,193 (11)	35 (0.6)	14 (0.2)	22 (0.4)	6,122 (99)	61 (48–74)	99.8 (99.6–99.9)	71 (57–83)	99.6 (99.5–99.8)
2016	5,676 (10)	24 (0.4)	15 (0.3)	20 (0.4)	5,617 (99)	55 (39–70)	99.7 (99.6–99.9)	62 (45–77)	99.6 (99.5–99.8)
2017	,5447 (10)	33 (0.6)	12 (0.2)	19 (0.3)	5,383 (99)	63 (49–76)	99.8 (99.6–99.9)	73 (58–85)	99.6 (99.5–99.8)
2018	6,465 (11)	35 (0.5)	21 (0.3)	23 (0.4)	6,386 (99)	60 (47–73)	99.7 (99.5–99.8)	62 (49–75)	99.6 (99.5–99.8)
1 year after primary TKA by sex									
Male	22,752 (40)	158 (0.7)	74 (0.3)	111 (0.5)	22,409 (98)	59 (53–65)	99.7 (99.6–99.7)	68 (62–74)	99.7 (99.6–99.8)
Female	33,553 (60)	125 (0.4)	65 (0.2)	96 (0.3)	33,267 (99)	57 (50–63)	99.8 (99.8–99.8)	66 (59–73)	99.4 (99.2–99.6)
1 year after primary TKA by Charlson Comorbidity Index level									
Low (0)	4,156 (7.4)	40 (1)	26 (0.6)	30 (0.7)	4,060 (98)	57 (45–69)	99.4 (99.1–99.6)	61 (48–72)	99.3 (99.0–99.5)
Medium (1-2)	35,417 (63)	133 (0.4)	73 (0.2)	108 (0.3)	35,103 (99)	55 (49–62)	99.8 (99.7–99.8)	65 (58–71)	99.7 (99.6–99.7)
High (≥3)	16,732 (30)	110 (0.7)	40 (0.2)	69 (0.4)	16,513 (99)	61 (54–62)	99.8 (99.7–99.8)	73 (66–80)	99.6 (99.5–99.7)
1 year after primary TKA by prosthetic type									
Monoblock	1,154 (2.3)	26 (2.3)	19 (1.6)	58 (5.0)	1,051 (91)	31 (21–42)	99.8 (99.6–99.9)	58 (42–72)	99.3 (99.6–99.7)
Modular	48,151 (98)	257 (0.5)	120 (0.2)	149 (0.3)	47,625 (99)	63 (58–68)	99.7 (99.7–99.8)	68 (63–73)	99.7 (99.6–99.7)

Abbreviations: BMI = body mass index; DKR = Danish Knee Arthroplasty Registry; IQR = interquartile range; DNPR = Danish National Patient Registry; HAIBA = Healthcare-Associated Infections Database (surveillance algorithm based on data from the DNPR and MiBa); MiBa = Danish Microbiology Database; TKA = total knee arthroplasty; PJI = periprosthetic joint infection.

#### Identification of revisions due to PJI

In the DKR, a revision due to PJI was identified when a primary TKA was followed by revision due to “deep infection” either “verified by microbiology” or “suspected” reported by the surgeon immediately after revision surgery. If multiple causes including PJI were reported, PJI was considered the main cause of revision.

In HAIBA, a revision due to PJI was identified when a primary TKA was followed by “any revision” as well as a minimum of 2 positive microbiology tests (out of at least 3 samples) from MiBa revealing the same microorganism. Biopsies taken between 24 hours before to 48 hours after revision surgery were considered. These criteria correspond to the PJI definition [13] and follow HAIBA’s automated algorithm [18].

### Covariates

We used the CRS to obtain median age (years with interquartile range [IQR]) and sex. Data from the DKR allowed us to obtain the year of index surgery (2010 through 2018), time from index surgery to revision due to PJI as registered in the DKR (within 3 months and 1 year), Body Mass Index (BMI with IQR), and prosthetic type (monoblock without replaceable liner or traditional modular TKA with replaceable liner [25]).

From HAIBA we assessed the time to revision due to PJI (within 3 months and 1 year).

From the DNPR we retrieved the procedure codes of “any revision,” the history of comorbidity before the index date, as measured by the Charlson Comorbidity Index (CCI) [26] based on both primary and secondary discharge diagnosis codes, as well as hospital admissions and outpatient clinic visits. We defined 3 levels of comorbidity: low (CCI score = 0), medium (CCI score 1–2), and high (CCI score ≥ 3). We chose a 10-year lookback period based on previous evidence and data availability [27].

### Statistics

We examined the validity of data on revision due to PJIs recorded in the DKR using the HAIBA algorithm as a reference standard by calculating and describing the true positive, false positive, false negative, and true negative cases. The median age and median BMI on the index date were tabulated for each of the 4 validity groups. We then calculated 4 measures of validity: sensitivity, specificity, positive predictive value (PPV), and negative predictive value (NPV) with a 95% confidence interval (CI) overall and by year of TKA, sex, CCI level, and prosthetic type.

The false negative group represents cases of revision due to PJI not identified by the DKR. To evaluate any misclassification of patients in the false negative group, we first listed the procedure code from the DNPR that combined with the microbiology result led to the HAIBA diagnosis of PJI and then listed the revision cause recorded in the DKR if patients were registered with a revision other than PJI. In case of any misclassification, we regrouped the misclassified cases and recalculated an overall best-case sensitivity.

We calculated the net incidence of revision due to PJI using the Kaplan–Meier (KM) method with a 1-minus-survival approach, where censoring occurred at non-PJI revision and death. This approach offers insights from a “surgeon’s perspective,” which can be valuable for allocating resources for revisions due to PJI. To provide a “patient’s perspective,” we also computed the cumulative incidence of revision due to PJI with the Aalen–Johansen method considering non-PJI revision and mortality as competing risks [28].

The contents of this paper follow the Strengthening the Reporting of Observational Studies in Epidemiology guidelines and the Reporting of Studies Conducted using Observational Routinely-collected Data guidelines [29]. Analyses were performed using SAS V.9.4 (SAS Institute, Cary, NC, USA) and R V.3.6.1 (R Foundation for Statistical Computing, Vienna, Austria).

### Ethics, funding, and disclosures

As this study was non-interventional, no ethical approval was required. The study was reported to the Danish Data Protection Agency through registration at Aarhus University (Record number AU-2016–051–000001, sequential number 880). Data were obtained specifically for this project under permissions granted by relevant Danish data authorities who own the data. This study was supported by the Health Research Fund of the Central Denmark Region and the Research Fund of the Department of Clinical Medicine, Aarhus University, Denmark. The funding sources were not involved in the design, analysis, or writing process. The authors report no conflict of interest. Complete disclosure of interest forms according to ICMJE are available on the article page, doi: 10.2340/17453674.2024.40358

## Results

Of the included 56,305 primary TKAs, 1,019 were identified in the DKR with a first-time revision within 1 year of the index surgery, whereas 1,122 were identified in HAIBA with a first-time revision based on the DNPR procedure codes (Figure 1).

Using HAIBA as a reference standard, first-time revision due to PJI in the DKR had a sensitivity of 58% (CI 53–62), correlating to an underreporting of 42% (Table 2). We found a specificity of 99.8% (CI 99.7–99.8), a PPV of 67% (CI 62–72), and an NPV of 99.6% (CI 99.6–99.7). For early revisions due to PJI within 90 days of the index date, the sensitivity was 53% (CI 48–58), the specificity was 99.9% (CI 99.9–99.9), the PPV was 75% (CI 69–80), and the NPV was 99.7% (CI 99.6–99.7). The sensitivity fluctuated by year of primary TKA and varied from 41% (CI 29–54) in patients treated in 2010 to 68% (CI 52–82) in 2012. In the strata defined by prosthetic type, the sensitivity of the modular TKAs was 63% (CI 58–68) and that of the monoblock TKA was 31% (CI 21–42). In the strata of sex and CCI level, the estimated 4 measures of validity were similar to the overall estimates. 

The 4 groups (true positive, false positive, false negative, and true negative) had similar characteristics across age, BMI, distribution of sex, and CCI group. This was not the case for the prosthetic type, which was unevenly distributed between the 4 groups, with 58 cases with a monoblock prosthesis in the false negative group representing the highest proportion (Table 2).

9 cases were classified as “Reoperation for superficial infection after surgery on the knee or lower leg.” In the DKR 43 (21%) out of 207 false negatives were registered with a different revision cause, mainly in the “other” category (n = 29) (Table 3). This was the case for 14% (26 of 183) of the false negatives within 3 months of surgery.

**Table 3:** Knee-related revision procedure codes from the DNPR used by HAIBA and revision causes other than PJI in the DKR among the 207 false negatives. Values are count

**Knee-related revision procedure codes ^a^ from the DNPR used in HAIBA**	
KNGW69 Reoperation for deep infection after surgery on the knee or lower leg	67
KNGS19 Incision and revision for infection in the knee joint	48
KNGS49 Incision and revision for infection in the knee joint with instillation of medication	20
KNGC Secondary insertion of a prosthesis in the knee joint	20
KNGA12 Open exploration of soft tissue in the knee joint	
KNGF02 Open synovectomy of the knee joint	17
KNGU1 Removal of all components of the total/lateral part of the knee replacement prosthesis	13
KNGW59 Reoperation for superficial infection after surgery on the knee or lower leg	9
KNGS29 Incision and revision for bone infection in the knee joint or lower leg	8
KNGS59 Incision and revision for bone infection in the knee joint or lower leg with the instillation of medication	
KNGS99 Other operation for infection in the tendon, joint, or bone in the knee or lower leg	
KNGW89 Reoperation for deep bleeding after surgery on the knee or lower leg	5
KNGW99 Other reoperation after surgery on the knee or lower leg	
Total	207
**Revision causes in the DKR other than PJI**	
Other	29
Instability	7
Aseptic loosening ^b^	–
Pain ^b^	–
Total	43

a Danish version of Nordic Medico-Statistical Committee (NOMESCO) surgical procedures.

b Numbers less than 5 cannot be reported due to Danish law.

Abbreviations: see Table 2.

In our sensitivity analyses, we reclassified the 9 cases of superficial PJI from the false negative to the true negative group. This reclassification led to an increase in sensitivity from 58% to 59%. When considering only the modular prostheses, to avoid any case of DAIR of a monoblock prosthesis without an exchangeable line (and henceforth not mandatory registration with the DKR) the sensitivity reached 63% (see Table 2). Combining reclassification of the 9 cases and only modular prostheses resulted in a best-case sensitivity of 64% for revision due to PJI in the DKR.

**Figure 2:**
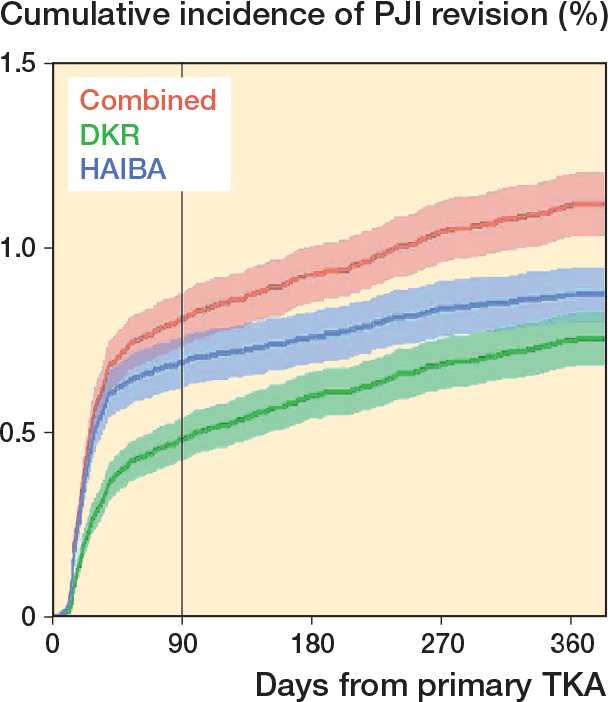
Cumulative incidences of PJI after TKA in percentages with 95% confidence intervals, according to the DKR, HAIBA, and DKR/HAIBA combined. For abbreviations: see footnote Table 2.

422 revisions due to PJI were recorded in the DKR (true positive or false positive), 490 were registered by HAIBA, and 629 revisions due to PJI in either the DKR or HAIBA within 1 year after the index surgery. This corresponds to a first-year net incidence of revision due to PJI of 0.8% in the DKR, 0.9% in HAIBA, and 1.1% when combining the DKR and HAIBA (Figure 2). Taking non-PJI revision and death into account as competing risks did not alter these results.

## Discussion

We assessed the validity of revision due to PJI after TKA recorded in the DKR against HAIBA as a reference and calculated the cumulative incidences within 1 year of TKA according to the DKR, HAIBA, and DKR/HAIBA combined. We found a sensitivity of data on revision due to PJI recorded in the DKR (vs. HAIBA) of 58% from 2010 through 2018, correlating to an underreporting of 42%. Accounting for possible misclassifications, the best-case scenario suggests an underreporting of 36%. 33% of revisions due to PJIs recorded in the DKR were culture-negative according to MiBa. Most false negatives were found within the first 3 months after the index TKA and were caused by non-reporting to the DKR rather than misclassification. When combining data from the DKR and HAIBA we calculated a cumulative incidence of revision due to PJI within 1 year of primary TKA at 1.1% (0.8% in the DKR and 0.9% in HAIBA).

Previous research has revealed that national arthroplasty registries in general are prone to underreport revision due to PJI [9-12], as also shown in the present study. PJI revision of knee arthroplasties is underreported by 13% in Finland [9] compared with the Hospital Discharge Registry; by 33% (hip) and 44% (knee) in Sweden [10,11] compared with the Swedish Prescribed Drug Register and subsequent medical record reviews; by 37% (hip and knee) in New Zealand [12] compared with local hospital datasets; and by 40% (hip) in Denmark [4] compared with microbiology, laboratory, and prescription data. The low sensitivity of revision due to PJI in arthroplasty registry data needs to be considered when interpreting findings based solely on these registries.

There are several possible explanations for the underreporting of revisions due to PJI in the DKR. A study of the Swedish Hip Arthroplasty Register [10] showed that habit or information available to the orthopedic community could play a role. Revisions due to PJI also often occur earlier than revisions for other causes. Thus, ambiguity regarding reporting of DAIR revision surgery only involving synovectomy and modular exchange could be a contributing factor, as was also seen in the New Zealand Joint Registry [12].

In the Norwegian Arthroplasty Registry, the accuracy of surgeon-reported revision cause in cases due to PJI after THA was 87% [30], emphasizing the importance of systematic correction of the reported revision cause, when results from biopsies taken during revision surgery are known. In the present study, 21% of missed PJI cases were erroneously registered under other causes in the DKR. Nevertheless, a more significant challenge was non-reporting, necessitating the use of alternative data sources, such as microbiology data, for comprehensive detection.

In the false positive cases, the surgeons registered PJI as a revision cause in the DKR without a present or subsequent positive culture, indicating a culture-negative PJI. A recent review of culture-negative PJI after hip arthroplasty and KA found that culture-negative PJI accounted for 5–42% of all PJIs [31]. According to the consensus guidelines [32], a diagnosis of PJI can be given in the absence of a positive culture, if a cutaneous sinus tract communicating with the prosthesis is present in a combination of acute inflammation, positive histology, or intraoperative evidence of purulence. A culture-negative PJI is frequently encountered when antimicrobial agents are used before obtaining culture samples [33] or in the presence of biofilm formation [34].

88% of the false negative cases occurred within 3 months after the primary TKA. As surgeons report to the DKR immediately after TKA surgery, when microbiology results are unknown, we hypothesized that misclassification of revision cause would be a major problem. Such misclassification is widely thought to be a contributing reason for the underreporting of revision due to PJI in hip arthroplasty registries [30,35]. However, our results showed that non-reporting was a larger problem than misclassification.

Their difficulty in adequately capturing revisions due to PJI necessitates a reconsideration of the data capture strategies within arthroplasty registries. Steps toward improvement may include validation against microbiology data as part of the annual report and feedback to orthopedic departments, or ultimately incorporation of microbiology data into arthroplasty registries. The present study presents one option, in which an automated microbiology-based PJI algorithm was used to assess the validity of revision due to PJI in arthroplasty registries. This algorithm has the potential to be integrated into arthroplasty registries and to improve the validity of PJI data, making arthroplasty registries a more useful tool for PJI research.

### Strengths

Strengths of this study include its nationwide population-based study design with a large sample size and complete follow-up, thereby minimizing the risk of selection bias. Completeness of the registration of primary TKA in the DKR was very high during the study period [23], thus our results are likely representative of the entire Danish TKA population.

Our study included both PJI cases recorded in the DKR that were “suspected” and those that were “verified by microbiology.” This approach was used because the decision to proceed with revision surgery is made only given strong clinical suspicion of PJI. Our study period of 9 years enabled assessment of the validity on an annual basis, information relevant for the interpretation of temporal trends in PJI incidence. The inclusion of unique microbiology data from MiBa, which were linkable at the patient level, allowed us to overcome limitations observed in previous studies [9-12], in which accessibility to microbiology data was limited to medical reports and thereby precluded comprehensive analysis of large datasets. By including microbiology data collected by an automated algorithm, we have introduced a method for improving the validity of data on revision due to PJI in an arthroplasty registry.

### Limitations

Only PJI leading to revision is included as an outcome, excluding PJIs treated without surgery.

PJI definitions in the DKR and HAIBA each lack some elements of the internationally accepted definition of PJI [13]. In the DKR the surgeon registers the cause of revision immediately post-surgery considering various preoperative assessments available at the time, but with no knowledge of the result of the perioperative biopsies. HAIBA defines PJI based on procedure codes and microbiology results, omitting preoperative workups. Both definitions, while not comprehensive individually, together cover essential elements. This confirms that combining data sources is crucial to capture PJI-related revisions effectively in large datasets [36].

The PJI definitions in each registry could have led to misclassifications, even when used in combination.

The data from MiBa does not provide interpretations of findings as pathogenic or contaminant. Moreover, the HAIBA algorithm does not incorporate preoperative aspirates and excludes cases with fewer than 3 samples or only 1 positive sample, irrespective of the bacteria type.

A bone infection of the lower leg within 1 year of a same-side, non-related successful TKA might incorrectly be labeled as a PJI revision by HAIBA. However, this study spans all TKAs since the inception of MiBa in 2010, and only a few isolated cases could be affected by this rare combination of events.

HAIBA caused prosthetic type misclassification, as DKR registration mandates exchange, removal, or modification for PJI revisions. Cases of monoblock TKAs without exchangeable liners undergoing DAIR are exempt from mandatory DKR registration, and contributed to varied prosthetic type distributions in the validity groups. Excluding monoblock TKAs in the analysis has slightly improved the sensitivity.

Discrepancies in the laterality of primary TKA between DKR and HAIBA may have excluded cases due to index date and laterality criteria. Revision procedure laterality discrepancies could result in underestimating true positive PJI revisions.

### Conclusion

In conclusion, revision due to PJI in the DKR was significantly underreported, particularly during the initial 3 months following primary TKA. Notably, the PJI cases missed by the DKR were primarily due to non-reporting, rather than misclassification. The cumulative incidence of PJI revision within 1 year after TKA was highest when combining data from the DKR with microbiological data. By doing so, it is possible to enhance the capture of PJI revision in an arthroplasty registry setting.

## Notes

EK and MA linked the cohort and oversaw data management. MA analyzed the data and drafted the manuscript. All authors participated in the design of the study, interpretation of the results, and approved the final manuscript.

Handling co-editors: Marianne Westberg and Robin Christensen

*Acta* thanks Håvard Dale and Marjan Wouthuyzen-Bakker for help with peer review of this manuscript.
